# Gut microbial metabolites in cutaneous inflammation: shared mechanisms and therapeutic opportunities

**DOI:** 10.3389/fimmu.2026.1850282

**Published:** 2026-07-14

**Authors:** Biyu Hu, Lin Du, Danfeng Chu, Erwen Kou, Haixia Zhao, Baiping Dong, Bo Wang, Yuanjie Zhu

**Affiliations:** 1 Department of Dermatology, Naval Medical Centre, Naval Medical University, Shanghai, China; 2 Department of Dermatology, The First Affiliated Hospital of Naval Medical University, Shanghai, China; 3 Naval Medical Centre, Naval Medical University, Shanghai, China

**Keywords:** cutaneous inflammation, endocrine signaling, gut microbial metabolites, gut-skin axis, immune regulation, neural signaling

## Abstract

Cutaneous inflammation is influenced by systemic signals beyond the skin, and gut microbial metabolites contribute to skin homeostasis and inflammatory responses. Major classes of gut-derived metabolites, including short-chain fatty acids, tryptophan-derived compounds, and secondary bile acids, may shape cutaneous inflammation through immune, neural, and endocrine pathways. However, current research remains fragmented across metabolite classes and pathways, and cross-pathway interactions remain unclear. As a result, the relationship between metabolite disturbances and distinct inflammatory phenotypes remains incompletely understood. Atopic dermatitis and chronic spontaneous urticaria are used as representative examples of this variability. This review summarizes major metabolite classes, the pathways linking them to cutaneous inflammation, and current therapeutic strategies targeting these pathways. Therapeutic strategies targeting gut microbial metabolites include direct metabolite supplementation, microbiome-targeted strategies that modify metabolite output, and indirect host-directed interventions. Available evidence suggests that gut microbial metabolites may serve as potential therapeutic targets in cutaneous inflammation. However, current limitations include context-dependent effects, limited causal evidence, variable treatment response, and unresolved issues in delivery and tissue specificity.

## 1 Introduction

Cutaneous inflammation is influenced by genetic susceptibility, environmental exposure, epithelial barrier status, neuroendocrine activity, and immune regulation (1). Atopic dermatitis (AD) and chronic spontaneous urticaria (CSU) are common inflammatory skin diseases. Their recurrent symptoms impair quality of life and represent a substantial social and economic burden (1–3). In recent years, treatments such as dupilumab, lebrikizumab, and omalizumab have improved outcomes in some patients (4). However, treatment responses remain variable, relapse is common, and the upstream factors that maintain disease activity are still not fully understood (5, 6).

Gut microbial metabolites have received increasing attention as systemic modulators of skin inflammation (7). These metabolites are products of microbial metabolism and may reflect host-microbe interactions more directly than microbial composition alone (8, 9). Short-chain fatty acids (SCFAs), tryptophan-derived metabolites, and secondary bile acids are the major metabolite classes most often studied in skin inflammation (7, 8). These metabolites may affect epithelial barrier integrity, immune activation, neuroimmune signaling, and endocrine responses. Therefore, they may contribute to cutaneous inflammation and pruritus rather than simply serving as indicators of gut dysbiosis (10).

Current evidence remains fragmented. Many studies focus on a single class of metabolites or one signaling pathway, whereas cross-pathway interactions are still unclear (7, 8). This makes it difficult to explain how similar upstream metabolite changes may result in different disease features. Metabolite effects are often context dependent and vary with concentration, receptor profile, tissue state, and disease background (8). AD and CSU provide two useful examples of this variability. In AD, metabolite-related mechanisms are often discussed in relation to epidermal barrier dysfunction, type 2 inflammation, and itch-associated sensory signaling (11). In CSU, the available evidence points to a mast-cell-centered inflammatory context. This may involve FcϵRI-related activation, systemic inflammatory signals, and possible neuroimmune amplification (12).

**Figure 1:**
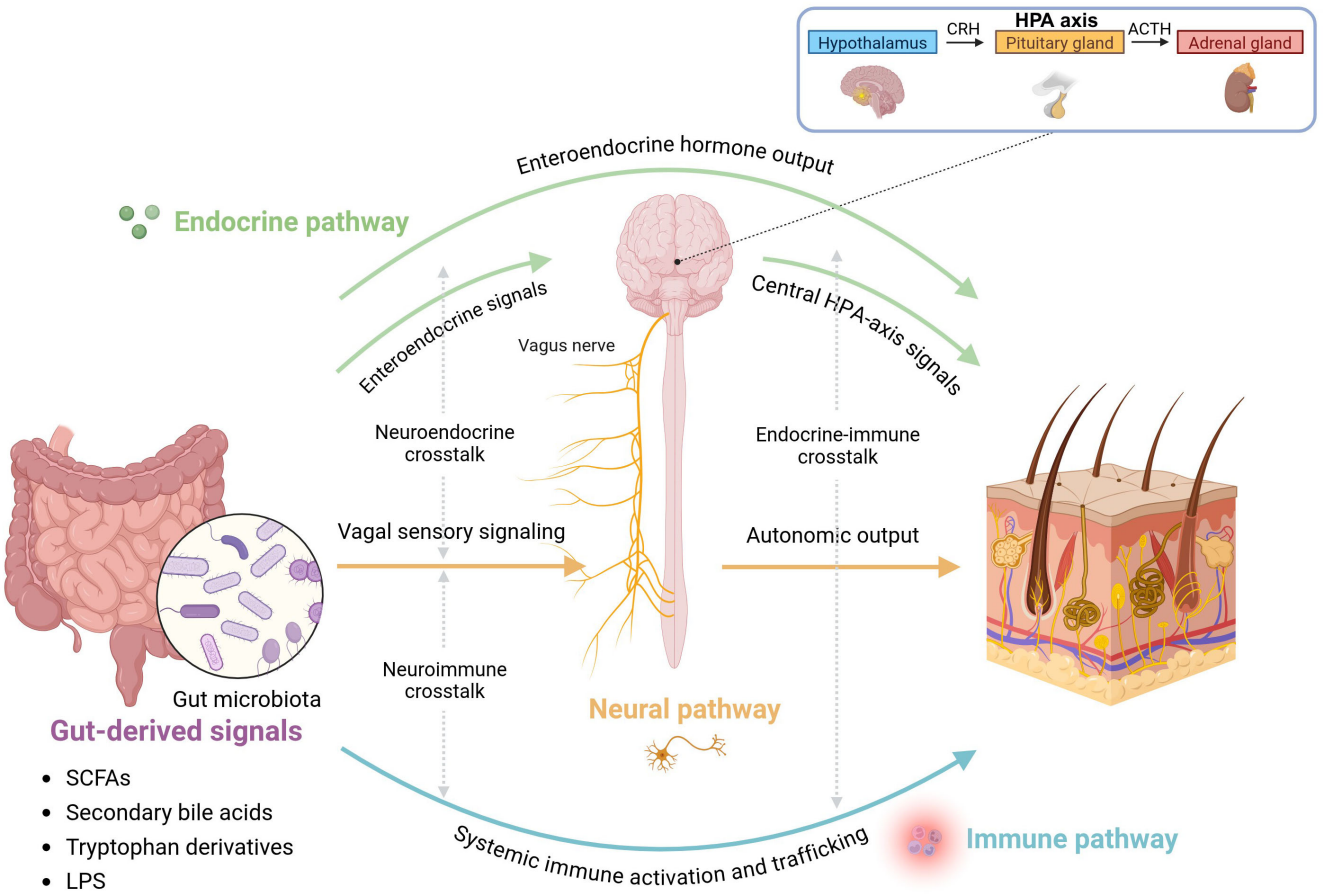
Integrated overview of gut-derived signals and cutaneous inflammation. Gut-derived signals, including SCFAs, secondary bile acids, tryptophan derivatives, and LPS, may influence cutaneous inflammation through immune, neural, and endocrine pathways. The immune pathway involves systemic immune activation and immune-cell trafficking. The neural pathway involves vagal sensory signaling and autonomic output. The endocrine pathway involves enteroendocrine hormone output and central HPA-axis-related signaling. Dashed arrows indicate possible crosstalk among these pathways, including neuroimmune, neuroendocrine, and endocrine–immune interactions. These pathways may jointly affect barrier function, cutaneous immune activation, pruritus, and inflammatory amplification. Created with BioRender.

This review focuses on gut microbial metabolites in cutaneous inflammation across immune, neural, and endocrine pathways (**Figure 1**). Rather than summarizing gut dysbiosis or individual metabolite classes alone, this review uses microbial metabolites as the central framework to integrate these pathways. AD and CSU are discussed as contrasting disease contexts to illustrate how metabolite-related mechanisms may vary across inflammatory skin diseases. It further summarizes current metabolite-oriented therapeutic strategies and discusses the main limitations affecting clinical application.

## 2 Immune pathways connecting gut dysbiosis and microbial metabolites to cutaneous inflammation

**Figure 2:**
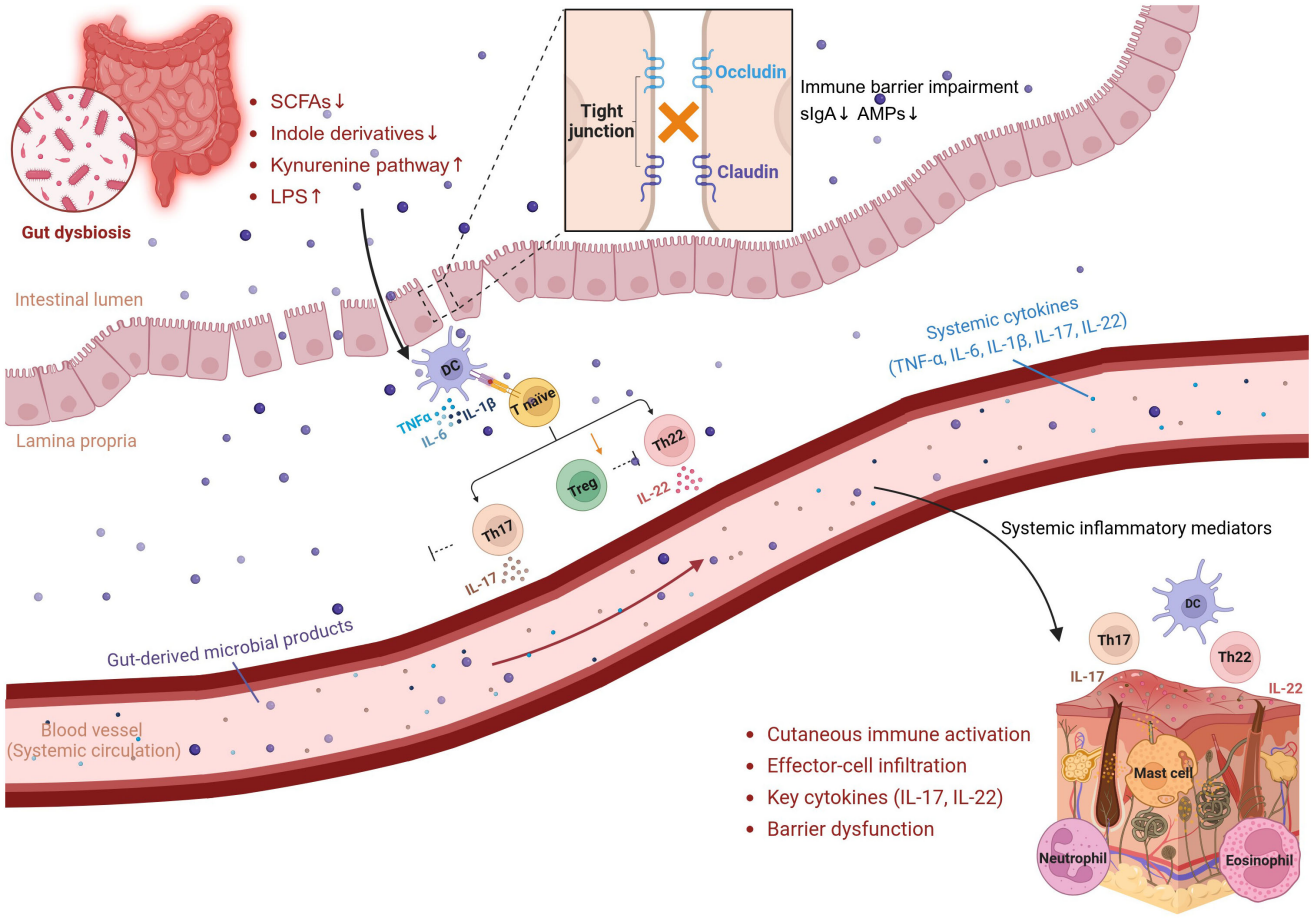
Immune pathways connecting gut dysbiosis and microbial metabolite changes to cutaneous inflammation. Gut dysbiosis is associated with reduced SCFAs and indole derivatives, increased kynurenine pathway activity, and increased LPS exposure. These changes may impair intestinal tight-junction integrity and mucosal immune barrier function, including reduced sIgA and antimicrobial peptides. Increased gut-derived microbial products may activate dendritic cells and promote systemic cytokine production, T-cell polarization, and immune-cell trafficking. These immune changes may contribute to cutaneous immune activation, effector-cell infiltration, barrier dysfunction, and local inflammatory amplification. Created with BioRender.

Immune pathways are crucial in mediating the effects of gut-derived microbial products on cutaneous inflammation (**Figure 2**). Intestinal barrier disruption increases systemic exposure to microbial products and inflammatory mediators. Altered metabolite profiles further disturb systemic immune regulation. These changes promote dendritic-cell (DC) activation, T-cell polarization, and cytokine release and increase susceptibility to inflammatory responses in the skin.

### 2.1 Intestinal barrier dysfunction and systemic immune activation

The intestinal barrier limits systemic exposure to luminal microbes, microbial products, and inflammatory mediators. It can be broadly divided into the intestinal mechanical barrier and the intestinal immune barrier (13). Disruption of either component may increase intestinal permeability and promote systemic immune activation.

The intestinal mechanical barrier is mainly formed by intestinal epithelial cells and tight-junction proteins (13). Tight-junction proteins connect adjacent epithelial cells and regulate paracellular permeability. When tight-junction integrity is impaired, luminal microbial products can more easily cross the epithelial barrier and enter the systemic circulation. This process may contribute to systemic inflammatory responses (14).

The intestinal immune barrier also plays an important role in limiting microbial invasion. Key components include antimicrobial peptides (AMPs) and secretory immunoglobulins (sIgs), especially secretory IgA (sIgA) (13). Reduced sIgA may weaken mucosal immune defense and increase bacterial translocation across the intestinal barrier (15). Local immune tolerance may also be impaired, further increasing inflammatory signaling.

Lipopolysaccharide (LPS) is a major microbial component involved in barrier-related immune activation (16). LPS exposure may reduce tight-junction protein expression and impair epithelial barrier integrity (17). Increased intestinal permeability can further enhance systemic exposure to LPS derived from Gram-negative bacteria (18). LPS activates Toll-like receptor 4 (TLR4) on immune cells and triggers NF-κB signaling (19). This can increase TNF-α, IL-1β, and IL-6 production and contribute to systemic low-grade inflammation. In this way, barrier dysfunction, LPS translocation, and inflammatory activation may form a feed-forward loop. This loop may lower the threshold for inflammatory activation in the skin (18).

### 2.2 Immune effects of microbial metabolites

#### 2.2.1 SCFAs in mucosal and systemic immune regulation

SCFAs are major microbiota-derived metabolites in mucosal and systemic immune regulation (20). They promote epithelial barrier integrity and mucin production, which helps maintain an intestinal environment that supports commensal stability (21). SCFAs can also regulate epithelial barrier function and mucosal immunity through G protein-coupled receptor (GPCR) signaling and histone deacetylase (HDAC) inhibition (21). In colonic epithelial cells, they regulate inflammatory signaling through GPCR-dependent pathways and alter chemokine and cytokine production (22). Acetate can reduce IL-6 and IL-8 production, and butyrate can exert anti-inflammatory effects *via* GPCR signaling and HDAC inhibition. These actions promote regulatory T cell (Treg) expansion and reduce T helper 17 (Th17) differentiation, which can decrease inflammatory activation (20, 23).

Germ-free (GF) mice show impaired nutrient absorption and altered intestinal morphology. Maturation of intestinal immune cells is also reduced, including intestinal intraepithelial lymphocytes, Th17 cells, and Tregs (23). These changes are accompanied by lower AMP production, reduced IgA secretion, and increased T helper 2 (Th2) cytokine signaling (24). GF mice have also been reported to lack several important fecal SCFAs (25). Treatment of GF mice with the butyrate-producing bacterium *Clostridium tyrobutyricum* improved tight-junction function. Oral sodium butyrate showed a similar effect (26). These findings suggest that the immune abnormalities observed in GF mice may be partly related to the absence of microbial fermentation products.

A proportion of intestinal SCFAs enters the portal circulation. After passing through the liver, these metabolites may reach the systemic circulation (20). This allows SCFAs to participate in systemic immune regulation. They can regulate adaptive immunity by influencing CD4^+^ T-cell differentiation and supporting CD8^+^ T-cell effector responses through metabolic and epigenetic mechanisms (21). In DCs, butyrate and propionate reduce the release of pro-inflammatory chemokines, including CXCL11, CXCL10, CXCL9, CCL5, CCL4, and CCL3. They also inhibit LPS-induced IL-6 and IL-12p40 expression (26). Through these systemic immune effects, reduced SCFA activity may contribute to systemic immune activation and increase susceptibility to cutaneous inflammation.

#### 2.2.2 Tryptophan metabolites and systemic immune regulation

Tryptophan metabolism is another pathway through which gut microbiota can modulate systemic immunity (27). In the intestine, dietary tryptophan is mainly metabolized through the indole, kynurenine (KYN), and serotonin (5-HT) pathways (28). The functions of indole metabolites are diverse and remain incompletely characterized. Some indole derivatives may strengthen the intestinal barrier, regulate T helper cell differentiation, and increase intestinal IL-22 production. Specific microbiota-derived indoles, including indole-3-acetic acid (IAA) and indole-3-propionic acid (IPA), may also affect systemic immunity. Many of these metabolites act as aryl hydrocarbon receptor (AhR) ligands and support immune-cell differentiation, barrier protection, and immune homeostasis (28, 29).

The KYN pathway also affects both innate and adaptive immunity, especially in macrophages, DCs, Tregs, and Th17 cells (29). The systemic KYN-to-tryptophan ratio is commonly used to assess the KYN pathway. A higher ratio usually indicates enhanced indoleamine 2,3-dioxygenase (IDO)-mediated tryptophan catabolism (30). Inflammatory signals can further promote this pathway by increasing IDO expression in immune cells (31). The IDO-KYN-AhR axis has context-dependent immune effects. In acute inflammation, it may help control excessive immune activation. In chronic inflammation, persistent activation of this axis may harm host cells in inflamed tissues and impair immune-cell function (32).

Serotonin (5-HT) is the major product of the 5-HT pathway. More than 90% of serotonin in the body is produced in the gut, mainly by enterochromaffin cells (ECs) (33). However, 5-HT is not a classical microbiota-derived metabolite. Gut microbiota may promote intestinal 5-HT synthesis from tryptophan by upregulating tryptophan hydroxylase 1 (TPH1), a key enzyme in 5-HT synthesis (34). 5-HT also regulates multiple innate and adaptive immune cell types, including DCs, macrophages, T cells, natural killer (NK) cells, B cells, and mast cells. It may either promote inflammation or exert protective effects, depending on receptor expression, cell type, and disease context (35).

### 2.3 Immune responses in the skin

#### 2.3.1 Gut-skin T-cell trafficking

Intestinal inflammation and altered systemic immune regulation can disrupt skin immune homeostasis. Merana et al. demonstrated that intestinal inflammation impaired adaptive tolerance to commensal microorganisms in the skin. Colitis increased the trafficking of gut microbiota-reactive CD4^+^ T cells to skin-draining lymph nodes and enhanced neutrophil infiltration in the skin. It also reduced commensal-specific Tregs. These changes were accompanied by cutaneous inflammation, whereas inhibition of lymphocyte trafficking restored cutaneous immune tolerance. Activated intestinal Th17 cells may circulate and contribute to inflammation in distal tissues, including the skin. Subsequent studies report that gut antigen-reactive T cells can migrate from the gastrointestinal tract and promote skin inflammation. Adoptive transfer of these cells induces cutaneous inflammatory reactions in naïve recipients, and genetic or homing receptor-targeted strategies that limit trafficking attenuate disease (36). These observations suggest that immune trafficking from the gut may affect cutaneous immunity, although current evidence is still mainly experimental in inflammatory skin diseases.

#### 2.3.2 Systemic cytokines and keratinocyte activation

Gut microbial signals also shape systemic cytokine profiles that directly influence the skin. Microbial signals promote DC differentiation and have been associated with increased circulating IL-17A and IL-22. These cytokines promote keratinocyte proliferation and neutrophil recruitment to skin (1, 37). Elevated IL-22 enhances expression of epidermal AMPs, including S100A7, S100A8, S100A9, and β-defensins. It also promotes production of neutrophil chemoattractants, including CXCL8, CXCL5, and CXCL1. Furthermore, IL-22 inhibits keratinocyte differentiation, impairs barrier repair, and increases matrix metalloproteinase (MMP) production, which promotes extracellular matrix degradation. In psoriasis, both circulating IL-22 levels and Th22 cell numbers are elevated and show a positive correlation with disease severity (37). In AD, systemic cytokine signals are more often associated with impaired barrier repair, epidermal dysfunction, and chronic itch. In CSU, systemic immune activation is more closely related to mast-cell priming, histamine release, and recurrent wheal activity.

#### 2.3.3 Effector cell activation and pruritus amplification

Systemic inflammatory mediators activate immune and stromal cells, including mast cells, eosinophils, neutrophils, and fibroblasts, and amplify cutaneous inflammation. Mast cells and eosinophils contribute directly to both inflammation and itch. Mast cells release mediators such as nerve growth factor (NGF). Eosinophils release granule proteins, including eosinophil cationic protein (ECP), eosinophil-derived neurotoxin (EDN), and eosinophil peroxidase (EPX), which contribute to tissue injury and local inflammatory amplification (38). Persistent systemic inflammation increases immune-cell recruitment to the skin and sustains local cytokine-driven inflammatory activity.

In the skin, systemic immune activation promotes effector-cell activation, cytokine amplification, and itch-related inflammation. These immune changes lower the threshold for persistent inflammatory activity. This suggests that immune regulation is central to the effects of gut microbial metabolites on cutaneous inflammation. However, the roles of individual metabolites and other dysbiosis-related signals may differ between diseases. The metabolite changes most closely related to particular inflammatory phenotypes also remain undefined.

## 3 Neural pathways connecting gut microbial signals to cutaneous inflammation

**Figure 3:**
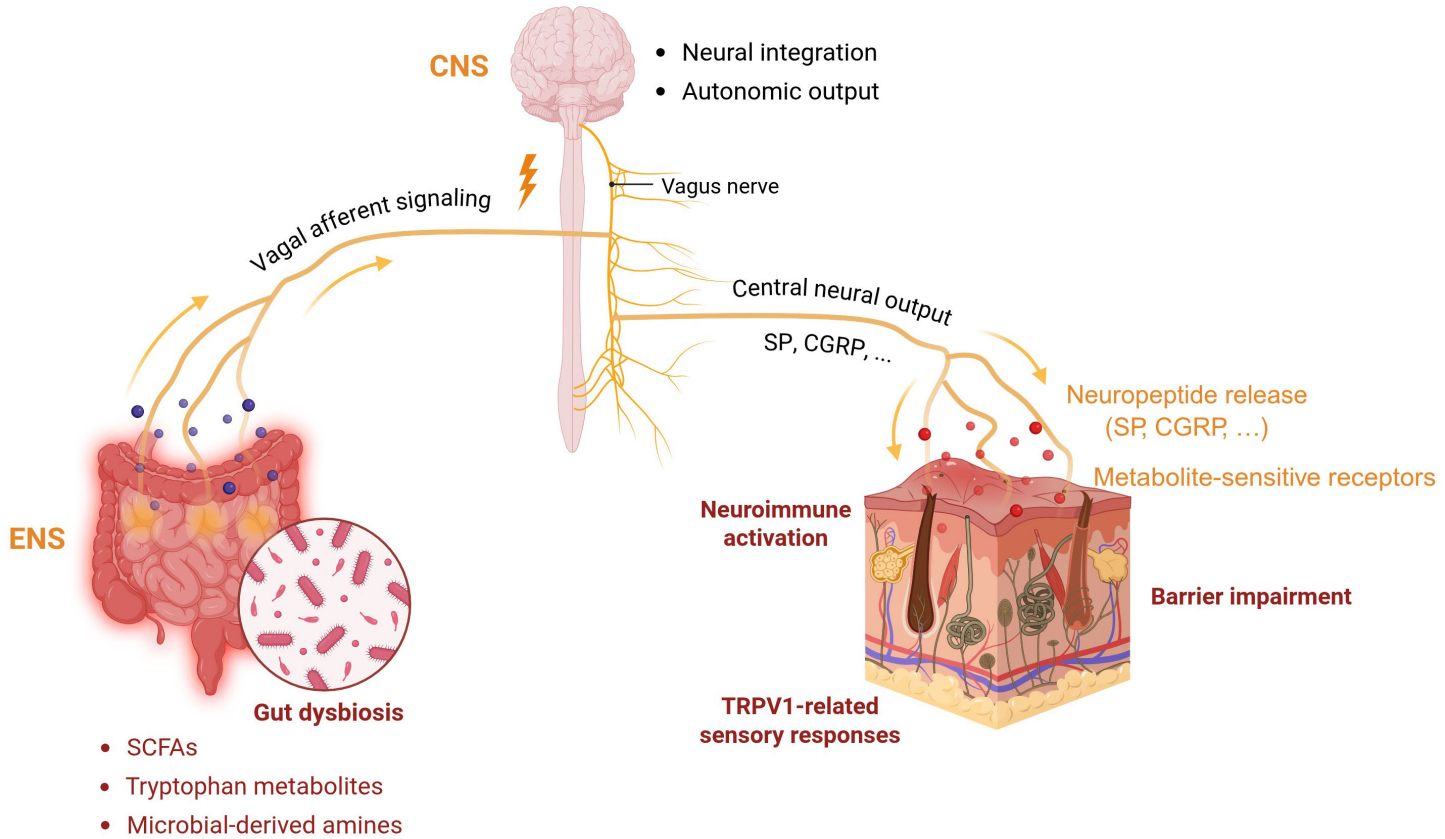
Neural pathways connecting gut microbial signals to cutaneous neuroimmune responses. Gut dysbiosis and microbial metabolites, including SCFAs, tryptophan metabolites, and microbial-derived amines, may influence enteric and vagal afferent signaling. These gut-derived neural inputs can be integrated in the central nervous system and translated into autonomic output. In the skin, sensory nerve activation and neuropeptide release, including SP and CGRP, may promote neuroimmune activation, TRPV1-related sensory responses, and barrier impairment. Direct evidence linking these neural pathways to inflammatory skin diseases remains limited. Created with BioRender.

Gut microbial metabolites can influence cutaneous inflammation through neural signaling. Gut-derived signals are sensed by vagal and enteric circuits, processed by autonomic and stress-related systems, and affect the skin through peripheral neuroimmune responses. This rapid signaling can contribute to itch, stress-related worsening of skin symptoms, and neuroimmune activation in the skin (**Figure 3**).

### 3.1 Vagal sensing of gut microbial metabolites

Signals from the gut reach the central nervous system (CNS) through the enteric nervous system (ENS) and vagal afferent pathways (7, 39). Approximately 80% of vagal fibers are afferent, providing a direct pathway for gut-derived signals to reach the CNS (40). The ENS senses intestinal signals and sends this information to vagal afferent pathways (41).

Gut microbial metabolites are involved in vagal gut-brain signaling (**Table 1**) (42, 43). Major classes include SCFAs, bile acids, tryptophan metabolites, biogenic amines, and other neuroactive microbial products (44). These metabolites can influence vagal afferent activity directly or indirectly through enteroendocrine signaling (42, 43). Gut microbes also produce γ-aminobutyric acid (GABA), 5-HT, dopamine, and acetylcholine (ACh), which can affect central signaling and immune responses (45, 46).

**Table 1:** Major gut-derived microbial signals in cutaneous inflammation.

Category	Source	Main host interface	Key sensors	Pathway-specific actions	Skin relevance
SCFAs	Fiber fermentation	Barrier-immune; enteroendocrine; neural	FFAR2/FFAR3; HDAC	Neural: vagal modulation; sensory sensitization. Endocrine: GLP-1/PYY/5-HT. Immune: Treg support; Th17 restraint.	Barrier maintenance; lower inflammation; itch modulation
Indole derivatives	Tryptophan metabolism	Endocrine-immune	AhR	Endocrine: GLP-1/5-HT regulation. Immune: AhR signaling; IL-22-related regulation.	Epidermal differentiation; barrier repair; immune regulation
Secondary bile acids	Primary bile acid conversion	Endocrine-immune; neural	TGR5; FXR	Endocrine: TGR5↑GLP-1; FXR↓GLP-1. Immune: inflammatory signaling. Neural: pruritus-related signaling.	Inflammation; pruritus-related responses; lipid homeostasis
LPS	Gram-negative bacterial wall	Barrier failure; immune activation	TLR4	Immune: cytokine induction; mast-cell priming.	Systemic inflammation; lower threshold for skin activation
Biogenic amines	Amino acid decarboxylation	Neural-immune	Histamine-related receptors	Neural: itch modulation. Immune: neuroimmune signaling.	Itch signaling; neuroimmune activation

Vagal signaling shaped by microbial metabolites may also influence inflammation through the cholinergic anti-inflammatory pathway (CAP). Signaling through the α7 subunit of the nicotinic acetylcholine receptor (α7nAChR) reduces systemic cytokine production (47). Chrna7 knockout models suggest that α7nAChR signaling is involved in microbiota-related vagal regulation of immune responses (48). The skin does not receive direct vagal innervation, but CAP may still shape cutaneous immunity through systemic inflammatory changes (47).

### 3.2 Central integration and autonomic output

Gut-derived neuroactive metabolites can influence central autonomic and stress-related processing *via* vagal or enteroendocrine signaling (40, 43). These inputs are processed in the CNS and can affect the skin through changes in autonomic output and neuroimmune activity (49, 50).

The CNS integrates visceral sensory input shaped by gut microbial metabolites and converts it into autonomic output (40, 41). Changes in autonomic output and stress reactivity can promote cutaneous immune activation and inflammatory flares (51). Altered central autonomic output is also associated with stress-related worsening of skin inflammation (51).

SCFAs are not classical neurotransmitters, but they can still affect neural signaling. They modulate vagal afferent activity and can cross the blood-brain barrier (BBB) (52). SCFAs also affect BBB integrity, neurotransmitter synthesis, neurotrophic signaling, and microglial function (53). Tryptophan metabolites and microbially derived amines can reach the CNS through vagal afferents, enteroendocrine signaling, or systemic circulation (9, 43). They can also alter stress responses and central sensory integration (52). These changes in autonomic output and stress reactivity can promote neurogenic inflammation and increase susceptibility to skin inflammation (50, 51).

### 3.3 Cutaneous neuroimmune responses influenced by microbial metabolites

The skin contains a sensory nerve network that contributes to neuroimmune regulation (54). Activation of cutaneous nerves releases neuropeptides and neurotransmitters, especially substance P (SP) and calcitonin gene-related peptide (CGRP). These mediators promote vasodilation, mast cell degranulation, leukocyte recruitment, and barrier disruption, amplifying local inflammatory responses (55, 56). Cutaneous sensory nerves are also activated through pruriceptive and neuroimmune mechanisms, including IL-31 receptor signaling and TRP-channel activation (38).

Gut microbial metabolites can modulate nerve-immune interactions. SCFAs, tryptophan metabolites, and microbial amines may influence sensory signaling and local neuroimmune activity (8). The effects of SCFAs are tissue- and context-dependent. Under stable barrier conditions, gut-derived SCFAs are more often associated with barrier protection and immune regulation (20). However, SCFAs may also affect neuronal excitability, neuropeptide release, and mast cell-neuron interactions (57). Under barrier injury, ongoing inflammation, or sensory hypersensitivity, SCFAs may enhance neuroimmune responses. Experimental evidence suggests that butyrate can influence mast cell-sensory neuron communication and TRPV1-related sensory responses under hypersensitive conditions (57). TRPV1 activation promotes CGRP and SP release, which can act on mast cells, macrophages, and T lymphocytes to amplify neuroimmune responses and neurogenic inflammation (58, 59). This supports a possible role for microbial metabolites in the regulation of cutaneous neuroimmune activity.

From vagal sensing to cutaneous neuropeptide release, neural signaling promotes sensory activation and local inflammatory amplification in the skin. However, most current evidence comes from separate studies on gut-brain communication, sensory pathways, and neurogenic inflammation, and direct evidence in the skin remains limited.

## 4 Endocrine pathways connecting gut microbial signals to cutaneous inflammation

**Figure 4:**
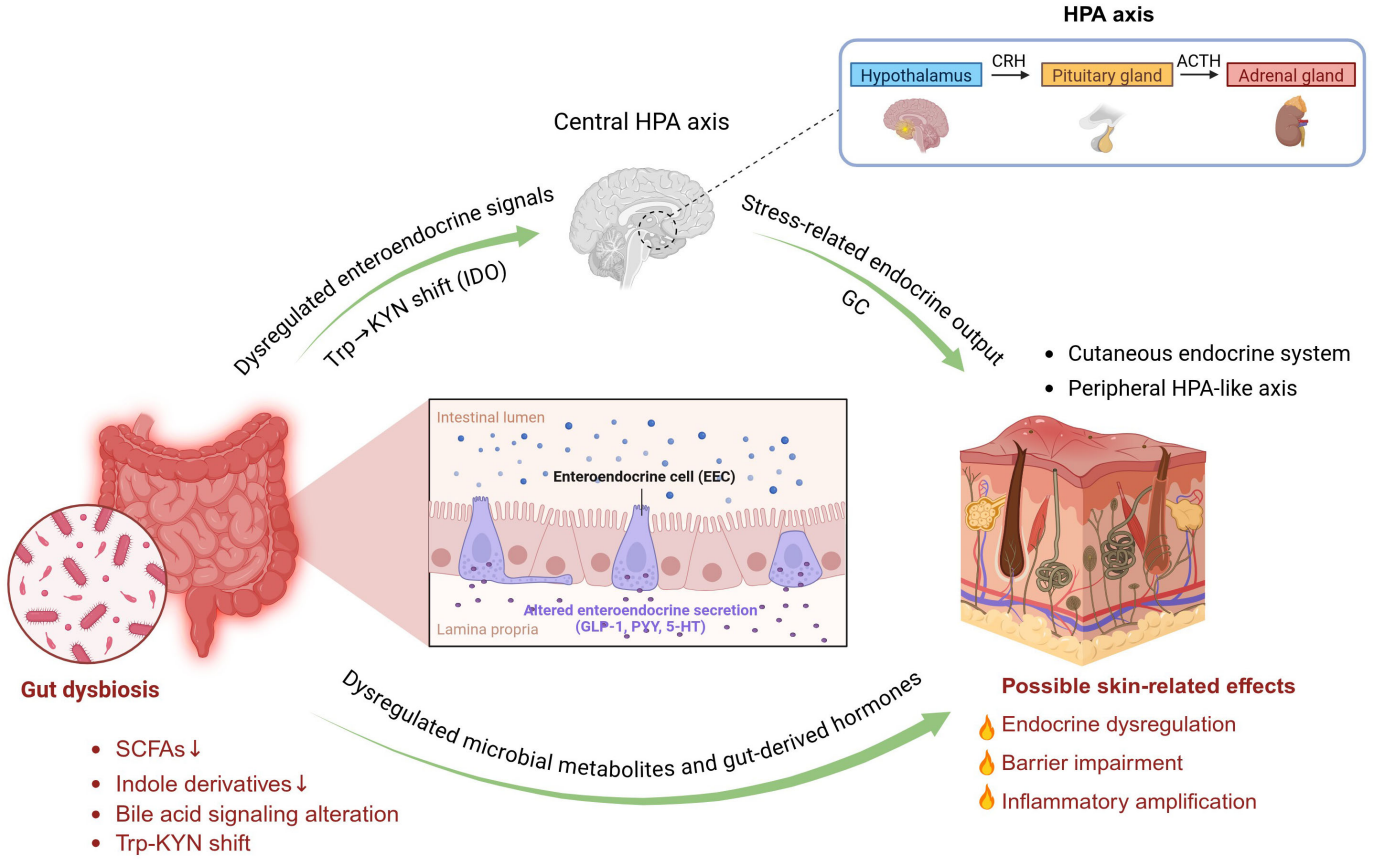
Potential endocrine pathways connecting gut microbial signals to cutaneous inflammation. Gut dysbiosis may alter SCFAs, indole derivatives, bile acid signaling, and tryptophan-kynurenine metabolism. These changes may regulate enteroendocrine cell activity and alter the secretion of GLP-1, PYY, and 5-HT. Gut-derived endocrine signals and stress-related HPA-axis output may further affect cutaneous endocrine regulation, including the local cutaneous endocrine system and peripheral HPA-like axis. These pathways may contribute to endocrine dysregulation, barrier impairment, and inflammatory amplification in the skin, but direct dermatology-specific evidence remains limited. Created with BioRender.

Gut microbial metabolites may influence cutaneous inflammation through two endocrine pathways (**Figure 4**). The indirect pathway begins with enteroendocrine sensing of luminal metabolites and continues through central neuroendocrine integration of gut-derived hormonal signals. The direct pathway involves metabolite-responsive signaling in the cutaneous endocrine system. This signaling may affect barrier function, inflammatory activity, and local hormone-related regulation. Compared with immune pathways, the endocrine evidence is less direct.

### 4.1 Intestinal enteroendocrine sensing of microbial metabolites

#### 4.1.1 Enteroendocrine cells and sensing mechanisms

The intestinal enteroendocrine system converts luminal microbial metabolites and related signals into hormonal outputs. This function is mediated by enteroendocrine cells (EECs), which detect luminal metabolites and secrete regulatory hormones. Open-type EECs contact the lumen and sense luminal signals through receptors including GPCRs, Toll-like receptors (TLRs), and taste receptors. After stimulation, these cells release hormones into the lamina propria or circulation. Some EEC subsets also form basal neuropod-like processes that support rapid communication with enteric neural circuits (60, 61). This sensing system converts luminal microbial metabolites into hormonal signals that influence systemic neuroendocrine activity (60, 61).

#### 4.1.2 Metabolite-driven hormone outputs

Several classes of gut microbial metabolites can regulate enteroendocrine hormone release. SCFAs can stimulate L cells to release glucagon-like peptide 1 (GLP-1) and peptide YY (PYY), mainly through SCFA-sensitive GPCRs such as FFAR2 and FFAR3 (42). SCFAs can also regulate enteroendocrine function through HDAC-related mechanisms (62). In ECs, butyrate can upregulate TPH1 and promote 5-HT synthesis and release (63–65). These hormone outputs may provide an indirect link between microbial metabolites and cutaneous inflammation.

Tryptophan metabolites also regulate enteroendocrine hormone release. Microbial tryptophan metabolism generates indole, tryptamine, indole-3-pyruvic acid (IPyA), and related derivatives (42). Some of these metabolites act through AhR, pregnane X receptor (PXR), or transient receptor potential (TRP)-channel-related pathways (42, 43, 66). They may influence GLP-1 release and 5-HT secretion (43, 67). These hormonal signals may affect downstream immune or neuroimmune responses.

Bile acids provide another metabolite-related endocrine signal. Gut microbiota can convert part of liver-derived primary bile acids into secondary bile acids, including lithocholic acid (LCA) and deoxycholic acid (DCA) (42, 67). These bile acids can regulate enteroendocrine secretion through farnesoid X receptor (FXR) and Takeda G protein-coupled receptor 5 (TGR5) signaling (42, 67). Through effects on GLP-1, PYY, and related hormonal outputs, bile acid signaling may provide upstream endocrine signals for later neuroendocrine integration.

### 4.2 Central neuroendocrine integration of gut-derived signals and skin-related effects

#### 4.2.1 Circulating microbial signals and HPA-axis modulation

Gut microbiota and their metabolites may influence hypothalamic-pituitary-adrenal (HPA)-axis function. Circulating microbial signals, including microbial antigens, cytokines, and metabolites, can contribute to systemic inflammatory activity and influence HPA-axis reactivity (14, 68). Increased intestinal permeability allows LPS translocation into the circulation and may affect HPA-axis activity through cytokine signaling across the BBB (14). GF animals show exaggerated HPA responses, anxiety-like behavior, and impaired cognition. These abnormalities are partly reversed by probiotic administration or fecal microbiota transplantation (FMT). This suggests that the microbiota may contribute to HPA-axis reactivity (52, 69).

Prolonged HPA-axis activation may further affect intestinal barrier and microbial homeostasis. Elevated cortisol suppresses sIgA production, downregulates tight-junction proteins, and increases intestinal permeability (70). Chronic stress is also associated with GC receptor dysfunction and reduced regulatory control of inflammation (71, 72). These changes may further increase systemic exposure to microbial signals and sustain HPA-axis reactivity.

#### 4.2.2 Microbial metabolites associated with HPA reactivity

Among microbial metabolites, SCFAs are the most extensively studied in relation to stress and HPA-axis activity. Higher systemic SCFA levels are associated with reduced cortisol responses to acute stress (52). SCFAs regulate gene expression through HDAC inhibition, with butyrate showing the strongest activity (73, 74). They have also been linked to neuronal homeostasis through effects on neurotrophic factors, including NGF and brain-derived neurotrophic factor (BDNF) (52, 75). In GF models, SCFAs have been reported to reduce excessive microglial activation and pro-inflammatory cytokine release (52). These findings suggest that SCFAs may reduce excessive HPA reactivity and stress-associated neuroinflammation.

Tryptophan metabolism also affects neuroendocrine regulation. Stress and inflammation increase IDO activity and shift tryptophan metabolism toward KYN, which is associated with neuroinflammation and increased HPA-axis activity (76, 77). Microbial indole derivatives can also activate AhR and may affect neuroendocrine function through this pathway (78).

#### 4.2.3 Central endocrine outputs relevant to skin inflammation

Stress exposure triggers corticotropin-releasing hormone (CRH) release from the paraventricular nucleus in the hypothalamus, followed by adrenocorticotropic hormone (ACTH) secretion and cortisol production (69, 79). This sequential release of CRH, ACTH, and cortisol forms the classical HPA-axis cascade. Cortisol and catecholamines modify cytokine signaling and immune cell responses in the skin (55). Stress-related endocrine responses have also been associated with impaired barrier function, altered keratinocyte differentiation, and increased inflammatory activity. These effects may contribute to flares in psoriasis and AD (49). Elevated cortisol may weaken epidermal barrier formation and antimicrobial defense (51). CRH-related sympathetic activation can alter cutaneous perfusion and sweat composition, which can modify the skin microenvironment (51, 80). These endocrine changes may affect barrier function, keratinocyte activity, and cutaneous immune responses. However, evidence for a direct pathway from gut microbial metabolites to HPA-axis activity and skin inflammation is still limited.

### 4.3 Direct cutaneous endocrine and metabolite-responsive pathways

#### 4.3.1 Cutaneous endocrine network and receptive capacity

The skin maintains an endocrine network that functions in parallel with central and enteric hormonal pathways. It produces hormones, neuropeptides, and cytokines locally and also responds to circulating signals shaped by gut microbial metabolites. The skin is both an endocrine target and a hormonally active tissue with independent regulatory capacity (49, 81).

Several cutaneous cell types produce CRH, ACTH, and GCs, including keratinocytes, melanocytes, fibroblasts, sebocytes, and mast cells. These cells also express the corresponding receptors (55). The skin also contains a peripheral HPA-like system (49, 82). This system responds to ultraviolet radiation, mechanical injury, irritants, and other stress-related inputs and regulates local inflammatory activity (55, 83). Increased CRH has been reported in serum and lesional skin in psoriasis (82, 83). Skin cells also produce α-melanocyte-stimulating hormone (α-MSH) and β-endorphin, further contributing to local endocrine regulation (82).

The skin is the major site of vitamin D3 synthesis. Through vitamin D receptor signaling, this pathway regulates keratinocyte differentiation, barrier formation, and immune responses in psoriasis and AD (84). The cutaneous endocrine network integrates local hormonal synthesis with signals derived from the circulation. These local endocrine features allow gut microbial metabolites to influence skin inflammation and barrier regulation.

#### 4.3.2 Metabolite-responsive pathways in cutaneous endocrine regulation

SCFAs modulate cutaneous endocrine-related inflammatory responses through both epigenetic effects and receptor-mediated signaling. In keratinocytes, SCFAs inhibit HDAC activity and suppress pro-inflammatory gene expression through epigenetic regulation (8, 74). SCFAs also reduce cytokine production, suppress NF-κB signaling, and activate GPR41/43. These actions may affect cellular metabolism and steroidogenesis in skin cells (8, 74).

Tryptophan-derived indole metabolites function as endogenous ligands for the AhR, which regulates epidermal integrity and immune balance. After ligand binding, AhR moves to the nucleus and forms a complex with AhR nuclear translocation protein (ARNT). AhR-ARNT signaling increases OVO-like 1 (OVOL1) transcription and promotes expression of barrier-associated proteins such as filaggrin (FLG) and loricrin (LOR). This supports terminal epidermal differentiation and barrier formation (85).

Bile acid signaling is another metabolite-responsive pathway relevant to the skin. Secondary bile acids such as LCA and DCA act through TGR5 and FXR in skin cells (86). These pathways affect inflammatory signaling, pruritus-related pathways, and lipid homeostasis. Changes in bile acid signaling have been reported in inflammatory skin diseases, and elevated bile acid levels may impair keratinocyte proliferation and barrier function (86).

In the skin, metabolite-responsive endocrine pathways may affect barrier function, inflammatory signaling, and local endocrine regulation. Evidence is relatively stronger for local cutaneous pathways, such as AhR-mediated epidermal differentiation, vitamin D signaling, and skin HPA-like responses. By contrast, the pathway from gut microbial metabolites to central HPA-axis activity and then to skin inflammation remains more indirect. Together, these findings suggest that gut microbial metabolites act through both local and systemic endocrine mechanisms. However, direct evidence connecting specific metabolites with endocrine responses and cutaneous inflammation is still limited. These pathways may influence disease pattern and inflammatory severity, but the evidence remains less direct than that for immune mechanisms.

## 5 Therapeutic strategies targeting gut microbial metabolites in cutaneous inflammation

Therapeutic strategies targeting gut microbial metabolites aim to correct dysbiosis-associated changes in microbial outputs that may influence cutaneous inflammation (18). These strategies include direct metabolite supplementation, microbiome-directed modulation, and host-targeted approaches (**Table 2**) (18, 87).

**Table 2:** Metabolite-oriented intervention strategies in cutaneous inflammation.

Strategy	Representative interventions	Main therapeutic aim	Main limitations
Direct metabolite supplementation	Postbiotics; metabolite analogs	Deliver bioactive metabolites directly	Poor bioavailability; rapid clearance; uncertain tissue delivery; context-dependent effects
Microbiome-directed modulation	Probiotics; prebiotics; synbiotics; dietary modification; FMT	Reshape microbial metabolite production	Variable response; heterogeneous evidence; safety and standardization
Host-targeted strategies	Psychological interventions; VNS; HPA-axis or circadian interventions	Alter metabolite output indirectly through host regulation	Indirect action; limited causal evidence; uncertain clinical benefit

### 5.1 Direct metabolite supplementation

Direct supplementation delivers bioactive molecules through postbiotics or metabolite analogs (88, 89). Current studies mainly focus on SCFAs, selected indole derivatives, and secondary bile acids. In experimental models, SCFAs support barrier integrity and reduce Th2 and Th17 responses, as well as mast cell activation (90). SCFA effects involve receptor-mediated signaling and epigenetic regulation. GPR41, GPR43, and PPARγ are involved, although some responses extend beyond these pathways (74, 91). In preclinical models, SCFA supplementation reduced inflammatory activity and improved barrier function in AD, psoriasis, and urticaria-related phenotypes (74). Indole-derived metabolites also show disease-dependent effects. In murine models, selected AhR-agonistic indole derivatives alleviated AD-like inflammation (85, 92). By contrast, indoxyl sulfate activated AhR in Th17 cells, remodeled chromatin accessibility, and amplified Th17-driven inflammation in psoriasis-like disease (93). Bile acid signaling through FXR and TGR5 contributes to inflammatory activity and pruritus in the skin (86). In a small number of clinical reports, oral or topical bile acid treatment reduced inflammation and pruritus in psoriasis and AD (86). Direct supplementation is limited by poor bioavailability, rapid clearance, uncertain tissue delivery, and context-dependent effects (94). Combination with microbiome-directed interventions may improve durability by supporting endogenous production (95). At present, most evidence remains preclinical.

### 5.2 Microbiome-directed modulation

Probiotics, prebiotics, synbiotics, dietary modification, and FMT can be used to alter microbial metabolite output (96–98). One month of oral *Escherichia coli* Nissle 1917 improved acne, papular-pustular rosacea, and seborrheic dermatitis. Gut microbiota showed increased *Bifidobacterium* and *Lactobacillus* and reduced pathogenic flora (99). In psoriasis models, selected strains of Bifidobacterium and Lactobacillus reduced skin inflammation and lowered IL-23- and Th17-related cytokines (100). Prebiotics, including inulin, resistant starches, fructo-oligosaccharides (FOS), and galacto-oligosaccharides (GOS), can increase SCFA production and may influence tryptophan and bile acid metabolism (97, 101), while synbiotics may yield more sustained effects (98). FMT has been explored in AD, but current evidence remains limited (102, 103). Some studies reported reduced disease severity after dietary intervention in acne, psoriasis, and AD, but treatment response varied between individuals (104, 105). These variable responses may reflect differences in baseline microbiota, diet, host immunity, disease subtype, and treatment exposure. Safety and standardization are also important issues, especially for FMT (106). The relation between clinical improvement and specific metabolite changes is still difficult to define.

### 5.3 Host-targeted strategies

Host-targeted strategies indirectly shift gut microbial metabolite output by acting on host regulators that shape gut physiology and microbial ecology, including psychological interventions, vagus nerve stimulation (VNS), and HPA-axis or circadian approaches (107–109). VNS can alter gut microbiota composition and SCFA-associated microbial outputs (107). Stress-reduction interventions can shift gut microbiota composition, including microbial features associated with SCFA production (108). Modulation of GC signaling or circadian regulation, including melatonin, can also influence SCFA-related metabolite profiles (109). These approaches are still indirect. Their effects on skin disease cannot be attributed to microbial metabolites alone. Key limitations include limited causal attribution, variable therapeutic effects, and uncertain durability. Future studies should clarify which host pathways regulate metabolite output and whether these changes improve clinical outcomes in skin disease.

### 5.4 Current clinical evidence for metabolite-oriented interventions

Current clinical evidence for metabolite-oriented interventions remains preliminary. Most available studies do not directly evaluate purified microbial metabolites. Instead, they use dietary interventions, probiotics, prebiotics, synbiotics, postbiotics, or FMT to modify gut microbiota and related metabolite output (18).

Several inflammatory skin diseases have been examined in clinical trials or small clinical studies. In AD, probiotics, prebiotics, synbiotics, postbiotics, and dietary supplements have been evaluated, and some trials reported improvement in skin severity scores (110). Psoriasis trials have mainly examined probiotics combined with standard treatment. Reported outcomes include disease severity, quality of life, and inflammatory markers (111). Oral bile acid supplementation has also been tested in psoriasis in earlier clinical studies (112). For chronic urticaria, probiotics have been combined with antihistamines in small clinical studies. Some studies reported changes in urticaria activity, wheal size, or attack frequency (18, 113).

These studies show growing interest in gut microbiota-based treatment, but direct metabolite-based interventions in skin diseases remain mostly experimental. Most clinical studies have not measured SCFAs, tryptophan metabolites, or bile acids as treatment-related endpoints. Future trials should combine clinical outcomes with microbiome profiling and targeted metabolomics.

Overall, metabolite-oriented therapy is still at an early stage in inflammatory skin disease. Current barriers include large differences in individual microbiota, limited reproducibility, and lack of standardized interventions. Reliable biomarkers for selecting suitable patients are also lacking. At present, these approaches should be considered investigational or supportive, rather than established treatments.

## 6 Disease-specific application of metabolite-oriented strategies

Metabolite-oriented strategies may need to be applied differently across skin diseases. In AD and CSU, immune, barrier, and neuroimmune mechanisms have more direct disease-related evidence. Endocrine-related pathways may also contribute, but current support is less direct and often comes from stress-response or gut-brain axis studies. Therefore, these pathways are discussed as possible modifiers rather than confirmed therapeutic targets.

### 6.1 Atopic dermatitis

AD is commonly characterized by epidermal barrier dysfunction, type 2-skewed inflammation, chronic pruritus, and altered microbial metabolite profiles (114, 115). Pruritus in AD is often driven by non-histaminergic signaling (114). Gut microbial metabolites influence barrier function, immune responses, and itch-related neural signaling. Among these altered metabolites, tryptophan-related compounds and GABA may contribute to itch-related neural and neuroimmune signaling in AD (116).

Individuals with AD often show lower SCFA levels than healthy controls (97). Reduced SCFA availability, particularly butyrate, may weaken Treg-associated immune regulation and permit stronger Th2-biased inflammation (117). Lower SCFA levels may disturb epigenetic regulation of epidermal barrier proteins and further impair barrier function (8). Tryptophan metabolism is also altered in AD. In a 2,4-dinitrofluorobenzene (DNFB) model, KYN increased while indole derivatives decreased relative to controls (92). Lower indole metabolite levels may reduce cutaneous AhR signaling and impair IL-22-related immune regulation and barrier repair (117).

Mechanistic studies also suggest that metabolite changes contribute to itch amplification. In murine AD models, propionate reduced dorsal root ganglion neuron responsiveness to multiple pruritogens, including IL-4. It also attenuated itch and alloknesis. This effect was largely independent of FFAR3 in dorsal root ganglion settings, suggesting mechanisms beyond canonical FFAR signaling (118). In addition, dysbiosis with increased intestinal permeability has been related to systemic exposure to gut-derived products such as LPS. In AD models, LPS aggravated itch through peripheral TLR4 signaling and increased itch severity without changing the initiating pruritogenic trigger (119).

In AD, SCFA and indole-derivative levels are reduced, whereas pro-inflammatory signaling is increased. Barrier repair, immune regulation, and itch control remain major therapeutic priorities. Metabolite-oriented strategies may support these priorities by improving epidermal barrier function, reducing type 2-skewed inflammation, and suppressing itch-related neural sensitization (92, 117, 118). Accordingly, direct supplementation and microbiome-directed modulation are preferred, whereas host-targeted approaches remain supportive.

### 6.2 Chronic spontaneous urticaria

Mast cell hyper-responsiveness, recurrent wheals, and pruritus are central features of CSU (**Table 3**). Metabolite dysregulation in CSU is more closely related to immune priming and flare susceptibility than to barrier failure (3).

**Table 3:** Disease-specific metabolite patterns, mechanistic features, and therapeutic priorities in AD and CSU.

Feature	AD	CSU
Core phenotype	Barrier dysfunction; Th2-skewed inflammation; chronic pruritus	Mast cell hyper-responsiveness; recurrent wheals; pruritus
Dominant metabolite pattern	↓ SCFAs; ↓ indole derivatives; altered tryptophan metabolism	↓ SCFA-producing taxa/SCFAs; ↓ beneficial indoles; ↑ LPS
Main mechanistic features	Impaired barrier repair; Th2-skewed inflammation; itch-related neural sensitization	Mast cell priming; inflammatory priming; neuroimmune amplification
Therapeutic priority	Barrier repair; immune regulation; itch control	Mast cell reactivity control; flare reduction; SCFA and indole restoration

Studies indicate reduced microbial diversity in CSU, with decreased SCFA-producing taxa and increased opportunistic pathogenic taxa (3, 12, 120). Reduced SCFAs may weaken Treg-mediated regulation and promote a pro-inflammatory environment. This metabolic shift may promote mast cell activation, increase degranulation, and contribute to CSU symptoms (12). Altered tryptophan metabolism in CSU may reduce indole derivatives and weaken AhR signaling, which could increase mast cell reactivity (3). Increased intestinal LPS in CSU may activate mast cells through TLR4 and enhance FcϵRI-related signaling, leading to IgE-driven degranulation and histamine release. This inflammatory priming also enhances SP-related neuroimmune signaling and aggravates pruritus and flare activity (120). Enteroendocrine mediators such as 5-HT and GLP-1 may also affect mast cell-related immune activity, but disease-specific evidence in CSU remains limited (3).

In CSU, mast cell hyper-responsiveness, inflammatory priming, and disruption of SCFA and indole regulation are central therapeutic targets (3, 12). Restoring SCFA and indole-related regulation may reduce mast cell reactivity and flare susceptibility, which is a key direction for metabolite-oriented treatment in CSU (120).

## 7 Discussion

Gut microbial metabolites may connect dysbiosis to cutaneous inflammation through immune, neural, and endocrine pathways. Changes in SCFAs, certain indole derivatives, secondary bile acids, and LPS-related signaling have been associated with skin inflammation. These alterations may affect barrier function, immune regulation, pruritus, and inflammatory activity. However, their effects are unlikely to be uniform across diseases. They may depend on barrier status, immune background, neuroendocrine context, and target-cell responsiveness. This supports a disease-oriented view of metabolite-related mechanisms and therapeutic development.

The limitations of current evidence differ across skin diseases. In AD, metabolite-related changes are closely related to epidermal barrier damage, type 2 inflammation, and itch. SCFAs and indole derivatives may therefore be more relevant to barrier repair, epithelial immune control, and sensory inflammation. However, it remains unclear whether these changes drive AD activity or occur as a result of inflammation, diet, barrier damage, or treatment. In CSU, the available evidence is more limited. The disease is less centered on barrier repair and more related to mast cell activation, FcϵRI-mediated responses, systemic inflammatory signals, and neuroimmune regulation. At present, direct evidence linking specific metabolites to mast cell activation thresholds, wheal formation, or treatment response is still insufficient. Therefore, metabolite-based approaches may need to be developed separately for AD and CSU, rather than applied in the same way across inflammatory skin diseases.

Despite recent progress, substantial mechanistic and translational gaps remain. First, many current studies show associations between metabolite changes and disease, but their direct causal role remains unclear. The key disease-related metabolites, targets, and signaling pathways have not been clearly identified. Metabolite effects can vary with dose, tissue context, and disease state. Future work will require metabolite tracking, multi-omics integration, and causal inference approaches. These approaches may help identify precise targets and signaling networks in skin cells and cutaneous microenvironments. Second, treatment effects show substantial inter-individual heterogeneity. Predictive markers of response across diseases remain limited. Disease- and subtype-specific models are needed to integrate microbiota composition, metabolite signatures, immune features, and host factors. Third, targeted delivery, quantitative control of metabolite exposure, and rational combination approaches still require further development to support disease-adapted strategies.

Metabolomics and microbiome studies are helping to identify disease-related metabolites and patient groups. However, these findings still need to be tested in specific skin diseases. Future studies should clarify which metabolite changes help drive disease and which are only secondary changes. They should also determine which targets can be used safely in treatment. This will be important before metabolite-oriented strategies can become reliable treatments for inflammatory skin diseases.
